# Prospective study of avian influenza transmission to humans in egypt

**DOI:** 10.1186/1471-2458-10-685

**Published:** 2010-11-09

**Authors:** Ghazi Kayali, Richard J Webby, Xiaoping Xiong, Lobna S Sherif, Esmat A El-Ghafar, Mohamed A Ali

**Affiliations:** 1 Division of Virology, Department of Infectious Diseases, St. Jude Children's Research Hospital, 262 Danny Thomas Place, Memphis, TN 38105, USA; 2 Department of Biostatistics, St. Jude Children's Research Hospital, 262 Danny Thomas Place, Memphis, TN 38105, USA; 3 Medical Division, the National Research Centre, Cairo 12311, Egypt; 4 Virology Laboratory, Environmental Research Division, the National Research Centre, Cairo 12311, Egypt

## Abstract

**Background:**

The highly pathogenic avian influenza (HPAI) H5N1 virus remains a public health threat and continues to cause outbreaks among poultry as well as human infections. Since its appearance, the virus has spread to numerous geographic areas and is now considered endemic in Egypt and other countries. Most studies on human H5N1 cases were conducted to investigate outbreak situations and were not designed to address fundamental questions about the epidemiology of human infection with H5N1 viruses. Our objective for this study is to answer these questions by estimating the prevalence and incidence rates of human cases and determine associated risk and protective factors in areas where H5N1 viruses are endemic.

**Methods/Design:**

We designed a 3-year prospective cohort study of 1000 individuals of various exposure levels to poultry in Egypt. At onset, we will collect sera to estimate baseline antibody titers against AI viruses H4-H16. Two follow-up visits are scheduled at 1-year intervals following initial enrollment. At follow-up, we will also collect sera to measure changes in antibody titers over time. Thus, annual prevalence rates as well as incidence rates of infection will be calculated. At each visit, exposure and other data will be collected using a specifically tailored questionnaire. This data will be used to measure risk and protective factors associated with infection. Subjects will be asked to contact the study team any time they have influenza-like illness (ILI). In this case, the study team will verify infection by rapid influenza A test and obtain swabs from the subject's contacts to isolate and characterize viruses causing acute infection.

**Discussion:**

Epidemiologic studies at the influenza human-animal interface are rare, hence many questions concerning transmission, severity, and extent of infection at the population level remain unanswered. We believe that our study will help tackle and clarify some of these issues.

## Background

Although the recent influenza pandemic was caused by a H1N1 virus, the pandemic threat of the highly pathogenic avian influenza (HPAI) H5N1 virus remains. Outbreaks of the virus in poultry and cases of human infection continue to occur. Severe economic loss is a usual consequence of H5N1 infection in poultry, as mortality and morbidity rates are high.

Exposure to poultry is the most important risk factor for humans becoming infected with HPAI viruses. Bridges et al. examined the sera of poultry workers in Hong Kong between the years 1997 and 1998 during which an outbreak of H5N1 was occurring in poultry. Using a nested case-control study design, they found that working in retail poultry operations, butchering poultry, feeding poultry, and preparing poultry for restaurants were factors significantly associated with seropositivity for antibodies against an H5 virus [1]. Similarly, in a case-control study of risk factors of infection with H5N1 viruses in Hong Kong in 1997, Mounts et al. found that cases were more likely to be exposed to live poultry than controls (OR = 4.5, 95% CI 2.1-21.7) [2]. In another case-control study, Dinh et al. reported that exposure to sick or dead poultry was a significant determinant of infection with H5N1 virus in a Vietnamese population in 2004 [3]. Also in Vietnam, Tran et al. noted that 9 of 10 H5N1 patients were exposed to poultry, 8 of whom had direct contact with birds [4]. In a population-based surveillance study in a rural area in Vietnam, a dose-response relationship between poultry exposure and flulike illness was noted: poultry in the household (odds ratio, 1.04; 95% confidence interval, 0.96-1.12), sick or dead poultry in the household but with no direct contact (odds ratio, 1.14; 95% confidence interval, 1.06-1.23), and direct contact with sick poultry (odds ratio, 1.73; 95% confidence interval, 1.58-1.89) [5].

Similar evidence comes from Thailand where H5N1 infections in birds and humans have been reported. Among 12 confirmed cases who came from areas where poultry were infected, 9 lived in households whose backyard chickens died, and 8 reported direct contact with dead chickens [6]. In another case-control study, Areechokchai et al. found that touching dead chickens was a significant risk factor for infection with H5N1 virus (OR = 29.0 95% CI 2.7-308.2). A case series of Turkish patients revealed that all 8 H5N1 virus infected patients had a history of contact with ill or dead chickens [7]. In a recent rero-epidemiologic study of 700 rural Cambodians, 18 individuals were sero-positive for H5N1 antibodies and were more likely to report swimming in community ponds [8].

All these studies took place in an outbreak situation and, by design, can only provide information on potential risk factors and perhaps the clinical profile of H5N1 human cases. Many fundamental questions remain unanswered: what are the true rates of infections among humans exposed to birds, do sub-clinical infections occur, and what are the true case-fatality rates. Such questions can be answered by conducting prospective epidemiological studies in geographic areas where H5N1 virus is endemic.

Initially appearing in Egypt in 2006, the H5N1 virus has since became endemic. In 2009 and 2010, Egypt reported the highest number of human infections with the virus and is currently the third country with the most reported cases following Indonesia and Vietnam [9]. Hence, Egypt is the ideal place to study the epidemiology of human infection with HPAI H5N1 viruses. Here we describe a prospective cohort study that aims to determine prevalence, incidence, and determinants of human infection with avian influenza (AI) viruses.

### Study Objectives and Hypothesis

This study has three primary objectives:

• To estimate prevalence of AI in poultry-exposed and non-exposed human populations.

• To estimate the incidence of AI in poultry-exposed and non-exposed human populations.

• To investigate risk factors associated with AI infections in occupationally-exposed poultry workers.

We hypothesize that by enrolling cohorts of individuals exposed and non-exposed to poultry and measuring avian influenza antibodies in their sera over a defined time period, we will be able to calculate true prevalence and incidence rates of AI infections. We also hypothesize that, by using a detailed exposure questionnaire, we will be able to determine risk and protective factors associated with infection.

By design, this study will also allow us to investigate patterns in transmission of AI to household contacts of AI cases as well as isolating and characterizing AI viruses causing acute illness in humans. This can be achieved by obtaining nasal swabs from cohort members reporting influenza-like illness (ILI) and their household contacts and testing for the presence of influenza A viruses.

## Methods/Design

### Study population and setting

The AI viruses under study are usually shed by live birds and transmitted to other birds through aerosolized secretions or environmental contamination. Thus we believe that individuals working with live birds are at an increased risk of infection due to their exposure to the environment and secretions of their poultry. Any individual who had an occupational contact with poultry in the previous five years will be considered exposed. Individuals working on poultry farms, poultry wet markets, or keeping limited numbers of poultry in their backyard, are considered exposed. Unexposed individuals must have no occupational exposure to poultry in their lifetime and must also not be exposed to poultry purchased from live bird markets. We will enroll 750 poultry exposed individuals from households, farms, and live poultry markets and 250 non-exposed controls.

**Figure 1:**
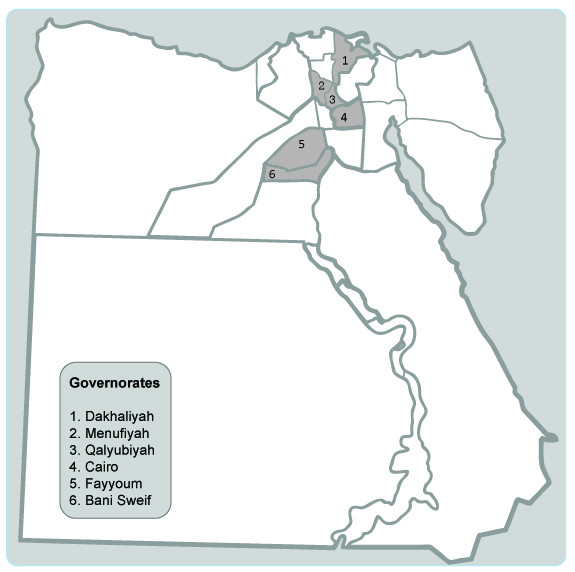
**Egypt's political map**. Governorates were enrollment sites are located are highlighted.

Exposed subjects will be enrolled at villages in Egypt where poultry production is located. The majority of the human cases of HPAI H5N1 infection in Egypt were located in the Nile Delta region in the North of the country. Our cohort of exposed subjects will be assembled from villages from 3 governorates in this area as well as 2 governorates south of Cairo where human cases of AI were reported. Hence, 5 villages will make up 5 field sites. From each village, we will enroll 150 individuals with exposure to poultry. The control group, i.e., individuals not occupationally exposed to poultry, will be enrolled from urban Cairo, the capital of Egypt. The study sites are highlighted in figure 1.

At each field site, we will invite poultry farm workers and poultry vendors at wet markets to participate. Following this, the sampling quota will be fulfilled by enrolling backyard farmers. All members of a household where poultry is raised will be enrolled. When the 150-participants quota is reached, enrollment at the specific site stops.

Controls will be enrolled in urban Cairo. Households will be selected from neighborhoods without live bird markets close to the location of our Cairo laboratory. All members of a household will be enrolled.

In this study, only children under 2 years of age will be excluded. Current data shows that almost all H5N1 cases were in individuals older than 2 years. This could be explained by the fact that these infants are not yet mobile and hence do not have access to poultry. Thus we will exclude this age group as the risks cannot be justified by scientific evidence. We expect that our sample will include children, females, and ethnic minorities reflecting the distribution of these groups in the general Egyptian population.

This study was approved by the St. Jude Children's Research Hospital (SJCRH) IRB (USA) and by the National Research Centre ethics committee (Egypt).

### Study design and procedures

**Figure 2:**
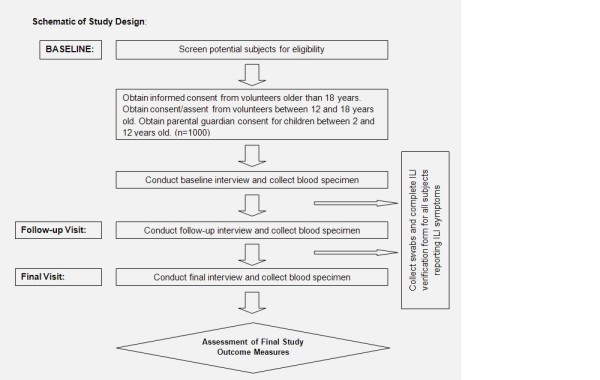
**Schematic of the study design**.

A schematic of the study design is presented in figure 2. We designed a 3-year prospective cohort study to compare individuals with occupational exposure to poultry with non-poultry exposed adult controls for evidence of incident and previous infections with AI viruses. At the start of this study, study staff will determine eligibility, obtain informed consent, and a blood sample will be obtained from study volunteers to establish baseline levels of antibodies against avian influenza types H4-H12. Subjects will be interviewed regarding their exposures, medical history, and behaviors using a close-ended questionnaire specifically tailored for this study. After one year, study subjects will be interviewed again to note any changes in exposure variables. At this time, another blood sample will be obtained and tested for any changes in influenza-specific antibody levels. The same procedures will be repeated at the final visit after another year. On follow up visits 1 and 2, study staff will interview the subject using a modified questionnaire that is designed to capture changes in baseline enrollment variables.

Study volunteers will be given specific instructions to note if they have any ILI symptoms and will be asked to contact study staff. If a subject is verified to have ILI symptoms, study staff will obtain 2 nasal swabs to be tested for the presence of influenza viruses. One of each of these swabs will be tested immediately for Flu A by using a rapid test kit. If any of these test positive, then study staff will obtain 2 nasal swabs from household contacts who are also enrolled in the study and complete the ILI verification form for each subject providing swabs. Furthermore, a blood sample will be collected from subjects with ILI symptoms for the purpose of harvesting peripheral blood mononuclear cells for immunological studies. The second swab will be tested by RT PCR; if influenza is present, further laboratory analysis will be performed to determine the type of virus present and the results will be confirmed at St. Jude Children's Research Hospital (SJCRH).

### Specimen handling

A trained phlebotomist will collect a tube of blood (7.5ml) in serum separator tubes. The blood will be allowed to clot at room temperature, then kept on ice until it arrives at the laboratory on the same day as collection where the specimens will be then centrifuged. Serum specimens will be aliqouted into multiple cryovials, labeled and preserved at -20°C until ready for laboratory study.

Swabs will be kept in tubes containing viral transport medium and kept on ice until received in the lab where they will be stored at -80°C until ready for laboratory study. Blood collected for peripheral cells will be collected in tubes containing heparin, then kept on ice until it arrives at the laboratory on the same day as collection where the specimens will be then processed. Reverse transcriptase (RT) PCR positive swabs for influenza A virus will be subtyped and will be shipped to SJCRH for confirmation and further processing.

### Laboratory analysis

Sera will be screened for human antibodies against AI viruses (types H4-H12) using a microneutralization assay at a dilution of 1:10. Sera that screen positive will be further studied through a microneutralization assay procedure and full titer determined.

Due to the potential cross-reactivity of antibodies against human influenza viruses confounding serological assays against AI viruses, we will also evaluate the sera against recently circulating human influenza viruses. Study sera will be examined for antibodies against human influenza types H1 and H3 using a hemagglutination inhibition procedure.

Swabs obtained from subjects reporting ILI symptoms and their household contacts will be screened for the presence of influenza A viruses by a rapid test. After arriving at the lab, specimens will be screened by RT PCR for presence of flu A by amplifying the M gene. Blood obtained from subjects reporting ILI symptoms will be used to collect peripheral blood mononuclear cells for immunological studies.

### Sample Size Calculation and Statistical Analysis

Population size estimates necessary to determine a difference of 19-29% in enrollment seroprevalence, with 5% significance level and 85-92% power, using STPlan (University of Texas) for comparing two binomial populations, revealed that 250 non-exposed controls and 600 exposed individuals will be needed.

Prevalence rates of infection with AI viruses will be calculated at baseline enrollment, follow-up, and final visits with 95% Confidence Intervals (CI). These rates will be stratified by age, gender, and exposure level.

Annual incidence rates will be assessed based on positive status defined as a 2-fold increase of antibody titers between baseline and follow-up and between follow-up and the final visit. Incidence rates will be calculated with their 95% CI and stratified by exposure level, gender, and age.

Risk or protective factors correlated with infection will be measured using the enrollment questionnaire. Changes in these factors over the study duration will be captured using the follow-up questionnaire. The odds ratios of prevalent infection with AI viruses and the relative risks of incident infection with AI viruses will be calculated based on each risk/protective variable. Multiple regression models will be used to determine risk and protective factors correlated with prevalence and incidence of AI infection among humans.

## Discussion

The circulation of pandemic H1N1 viruses in HPAI H5N1 endemic areas raises fears of emergence of a highly pathogenic virus efficient at human to human transmission. Given the zoonotic nature of influenza, such an event is most likely to occur at the human-animal interface. In addition, the scarcity of well-designed prospective epidemiological studies of zoonotic influenza transmission highlights the need for such studies and the importance of the one presented here.

## Competing interests

The authors declare that they have no competing interests.

## Authors' contributions

GK conceived of the study, is the study's lead epidemiologist, and will participate in coordination, data acquisition and analysis, and drafted the manuscript. RJW participated in study design, will supervise virus characterization, and helped to draft the manuscript. XX participated in the study statistical design and helped to draft the manuscript. LSS participated in the study design, helped to draft the manuscript, and will participate in coordination. EAE participated in the study design and helped to draft the manuscript. MAA participated in the study design, helped to draft the manuscript, and will help in coordination and supervise serological and molecular laboratory analysis. All authors read and approved the final manuscript.

## Pre-publication history

The pre-publication history for this paper can be accessed here:

http://www.biomedcentral.com/1471-2458/10/685/prepub
